# Neuromuscular characteristics of transcranial direct current stimulation over dorsolateral prefrontal cortex in patients with chronic low back pain: a randomized, double-blind, controlled trial protocol

**DOI:** 10.3389/fnhum.2025.1586257

**Published:** 2025-07-09

**Authors:** Xin Li, Wenzhao Liang, Zhicheng Li, Shijue Li, Yao Zu, Le Ge, Chuhuai Wang

**Affiliations:** Department of Rehabilitation Medicine, The First Affiliated Hospital, Sun Yat-sen University, Guangzhou, China

**Keywords:** chronic low back pain, muscle synergy, dorsolateral prefrontal cortex, primary motor cortex, functional near-infrared spectroscopy

## Abstract

**Background:**

Chronic low back pain (cLBP) is a prevalent condition associated with poor rehabilitation outcomes and high recurrence rates. Previous studies suggest that the number, structure, and network patterns of trunk muscle synergy may contribute to this condition. Our previous research mapped the neural representation of cLBP trunk muscles in the primary motor cortex (M1) and identified a disrupted M1-trunk muscle synergy pattern. Recent studies indicate that the dorsolateral prefrontal cortex (DLPFC) has a key role in the internal connectivity of cLBP patients when coping with chronic pain. This study aims to determine whether transcranial direct current stimulation (tDCS) of the DLPFC in cLBP patients can enhance the activity and functional connectivity of M1 and DLPFC, thus improving abnormal trunk muscle synergy.

**Methods:**

This study is a randomized, double-blind, controlled trial. Forty-eight individuals aged 20 to 55 years with cLBP will be randomly assigned to receive either (1) a 3-week DLPFC-tDCS intervention (*n* = 24) or (2) a 3-week M1-tDCS intervention (*n* = 24), administered four times per week for a total of 12 sessions. Clinical outcomes will be assessed at baseline, 3 weeks, and 6 and 12 months after randomization. Primary outcomes include pain intensity, disability, and scores on the Hamilton Depression and Hamilton Anxiety scales. Secondary outcomes include surface electromyography characteristics of trunk muscles, oxygenated and deoxygenated hemoglobin concentrations in the M1 and DLPFC, and functional connectivity between these two brain regions. These outcomes will be evaluated before and after the intervention. Effect sizes and a mixed-model repeated measures analysis of variance (2 groups × 4 time points) will be calculated.

**Discussion:**

The results of this trial will clarify the efficacy of DLPFC-tDCS in enhancing M1-DLPFC functional connectivity and improving trunk muscle synergy patterns. These findings will provide a theoretical foundation for developing new therapeutic targets for the treatment of cLBP.

**Clinical trial registration:**

https://www.chictr.org.cn, identifier ChiCTR2300078887.

## 1 Introduction

Low back pain (LBP) is a significant public health concern and a leading cause of disability worldwide, contributing to workplace absenteeism (Murray et al., 2012; Balagué et al., 2012). Chronic LBP (cLBP) is classified as nonspecific when no clear, identifiable cause is present and as chronic when it lasts longer than 12 weeks (Knezevic et al., 2021). An estimated 80% of adults have been reported to experience at least one episode of cLBP in their lifetime, with 40% developing a persistent form (Balagué et al., 2012; Zhou et al., 2024).

Various regions of the brain and spinal cord contribute to neuroplasticity in LBP, with research primarily focusing on the motor cortex (Brumagne et al., 2019). For instance, transcranial magnetic stimulation (TMS) over the primary motor cortex (M1) while monitoring trunk muscle activity has demonstrated changes in the stimulus–response profile of these muscles. This suggests neuroplasticity at M1 and changes in TMS-related trunk muscle activation (Tsao et al., 2008, 2011; Li et al., 2021). On the other hand, altered activation patterns in trunk muscles have been associated with movement avoidance behaviors due to perceived risk. These motor system adaptations may then affect spinal biomechanics. Previous studies indicate that during specific tasks, individuals with cLBP exhibit a reduction in trunk muscle synergy (Sung et al., 2019). Moreover, during symmetrical tasks, the trunk muscle synergistic network in cLBP patients has shown asymmetry (Rose-Dulcina et al., 2019; Lu et al., 2001). These results indicate that cLBP patients experience neuromuscular regulation dysfunction within the M1-trunk muscle synergy. Therefore, M1 has emerged as a potential therapeutic target for cLBP. However, recent reviews suggest that stimulation of M1 may not be effective in treating cLBP (Kandić et al., 2021). Furthermore, the dorsolateral prefrontal cortex (DLPFC) appears to play a key role in cognitive-behavioral processing and its association with chronic pain in cLBP patients (Kandić et al., 2021; Knotkova et al., 2021). This suggests that DLPFC could serve as an alternative target for top-down neural modulation in cLBP management.

Neuroadaptive changes in the central nervous system among cLBP patients are closely related to abnormal trunk muscle coordination patterns. Understanding the association between these trunk muscle functions and neurobiological changes is essential for optimizing treatment strategies. Non-invasive techniques, including electroencephalography, TMS, and functional magnetic resonance imaging (fMRI), have significantly contributed to advancing knowledge of brain function (Brumagne et al., 2019; Hodges and Danneels, 2019; Kenefati et al., 2023). However, their application in studying cortical dynamics during functional motor tasks is limited due to stringent environmental constraints, including the need for electromagnetic shielding, immobility requirements, and sensitivity to movement artifacts. To address these limitations, functional near-infrared spectroscopy (fNIRS) has emerged as a portable and non-invasive neuroimaging technique with unique advantages. fNIRS enables real-time monitoring of hemodynamic responses associated with neural activation through neurovascular coupling mechanisms (Ferrari and Quaresima, 2012). Moreover, fNIRS demonstrates greater resistance to environmental interference, allowing for the assessment of spatiotemporal characteristics of cerebral cortical activity during dynamic motor tasks.

Being another non-invasive technique, Transcranial direct current stimulation (tDCS) facilitates top-down neuromuscular regulation (Kandić et al., 2021). A fMRI study of healthy individuals showed that both M1-tDCS and DLPFC-tDCS increased the functional connection between M1-supplementary motor area (SMA), but only DLPFC-tDCS regulated the functional connection between M1-SMA-DLPFC. This indicates that M1-tDCS mainly regulates the functional connections of the motor sensory network, while DLPFC-tDCS can also regulate the functional connections related to cognition and emotion (Sankarasubramanian et al., 2017). However, whether tDCS effectively increases M1-DLPFC functional connectivity and improves abnormal trunk muscle synergy patterns in cLBP patients remains unclear.

This study primarily aims to assess the role of M1-DLPFC functional connectivity in regulating trunk muscle synergy patterns in cLBP patients. The primary hypothesis proposes that abnormal trunk muscle synergy in cLBP is associated with reduced M1-DLPFC functional connectivity. The secondary objective is to evaluate the effects of M1-tDCS and DLPFC-tDCS post-intervention and at follow-up in terms of (1) pain, (2) disability, (3) mental health, (4) surface electromyography characteristics of trunk muscles, and (5) the concentration of oxygenated and deoxygenated hemoglobin in the cerebral cortex of M1 and DLPFC. The secondary hypothesis is that DLPFC-tDCS is more effective than M1-tDCS in increasing M1-DLPFC functional connectivity and improving symptoms of depression and anxiety.

## 2 Methods and analysis

### 2.1 Study design

**Figure 1:**
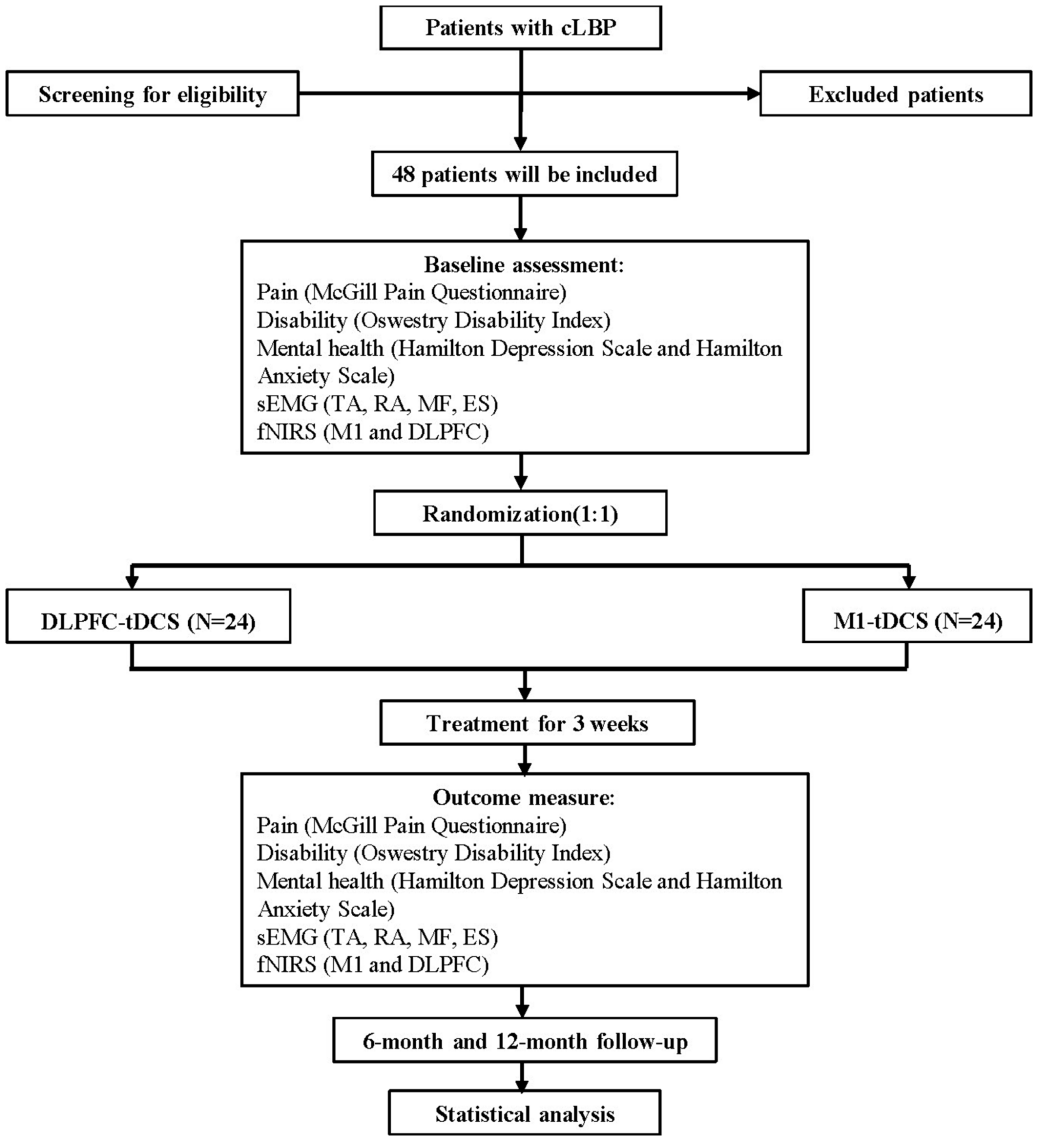
The study flowchart diagram. DLPFC, dorsolateral prefrontal cortex; ES, erector spinae; fNIRS, functional near-infrared spectroscopy; MF, multifidus; M1, primary motor cortex; RA, rectus abdominis; sEMG, surface electromyography; TA, transversus abdominis; tDCS, transcranial direct current stimulation.

This randomized, double-blind, controlled trial will be conducted at the Department of Rehabilitation Medicine, First Affiliated Hospital, Sun Yat-sen University, China. The trial will follow a blinded design in which assessors, therapists, and participants remain unaware of group assignments. Participants will be randomly assigned to one of two groups: (1) the Study Group receiving DLPFC-tDCS and (2) the Control Group receiving M1-tDCS. Assessments will be performed at baseline, 3 weeks, 6 months, and 12 months post-randomization, with the primary outcome evaluation occurring at 3 weeks. The study will be reported in accordance with CONSORT guidelines. The experimental flowchart is illustrated in Figure 1.

### 2.2 Ethics

The study protocol has been approved by the Ethics Subcommittee of the First Affiliated Hospital of Sun Yat-sen University ([2023]710) and is registered in the Chinese Clinical Trial Registry (ChiCTR2300078887). Participants have the right to withdraw from the study at any time.

### 2.3 Recruitment

Participants will be recruited through advertisements, outpatient clinics, and social media platforms. A designated research team member will conduct preliminary screening before study enrollment. The individual responsible for screening and assessments will remain blinded to the treatment group allocation. All eligible participants will receive detailed information about the study objectives and must provide written informed consent before participation. A screening log will be maintained to document non-recruited patients and reasons for exclusion.

### 2.4 Participant retention

All study components and timelines will be clearly explained to participants during the eligibility screening process to minimize attrition. This approach aims to prevent data loss due to scheduling conflicts. Moreover, participants will receive appointment reminders *via* text messages. Treatment attendance will be documented for each intervention session to further support participant retention.

### 2.5 Participants

#### 2.5.1 Inclusion criteria

1. Clinically diagnosed with cLBP, intermittent or persistent pain below the 12th rib of the lower back;
2. Chronic LBP lasting at least 12 weeks;
3. Reported pain intensity of 4–7 on the Visual Analogue Scale (VAS) at least once in the past week;
4. Age between 18 and 55 years;
5. Right-handedness; and
6. Both male and female participants.

#### 2.5.2 Exclusion criteria

1. Diagnosed or suspected serious spinal conditions (e.g., tumors, fractures, rheumatologic or inflammatory disorders, spinal infections);
2. Presence of nerve root impairment;
3. History of spinal surgery, including procedures outside the lumbar region or planned spinal surgery;
4. Pain related to pregnancy or structural deformities (e.g., scoliosis);
5. Current pregnancy;
6. Comorbid conditions that could limit participation in exercise programs, such as hypertension or cardiorespiratory disease;
7. Contraindications to tDCS, including the presence of a pacemaker, artificial metal heart valve, aneurysm clips (except titanium alloy), or other metal implants;
8. Ongoing physical therapy or participation in structured exercise programs;
9. Mental illness;
10. Current use of nonsteroidal anti-inflammatory drugs (NSAIDs) or planned initiation of NSAID therapy during the trial; and
11. Inability to tolerate the experimental procedures.

#### 2.5.3 Sample size

Based on the following formula and our preliminary experimental results of the primary outcome measures (VAS), with a significance level of *α* = 0.05, test power (1-*β*) = 0.9, a mean difference of 1.15, and a variance index of 1.48, it was determined that 20 participants per group are required. To account for an estimated attrition rate of 120%, a total of 48 cLBP participants will be recruited. To compare the effects after the intervention of the two groups, twenty-four right-handed healthy individuals with no history of cLBP were recruited.


$$N={\left[\frac{(t_{\alpha ∕2}+t_{\beta })S}{\delta }\right]}^{2}$$

### 2.6 Randomization and blinding

Forty-eight participants will be randomly allocated to either the DLPFC-tDCS group (Study Group) or the M1-tDCS group (Control Group). The randomization process will be conducted by a researcher who is not involved in participant recruitment, treatment, or assessment. This researcher will be instructed to keep the assigned interventions confidential from the participants, therapist, and other researchers until the study is completed. Randomization will be performed while employing the pseudo-random number generating RAND() function in Microsoft Excel, which produces random values between 0 and 1. Each generated number will then be ranked using the RANK() function in Excel, assigning a unique position to each value. The sorted random numbers will be assigned to groups, with values ranked 1–24 allocated to the Control Group and those ranked 25–48 assigned to the Study Group.

**Figure 2:**
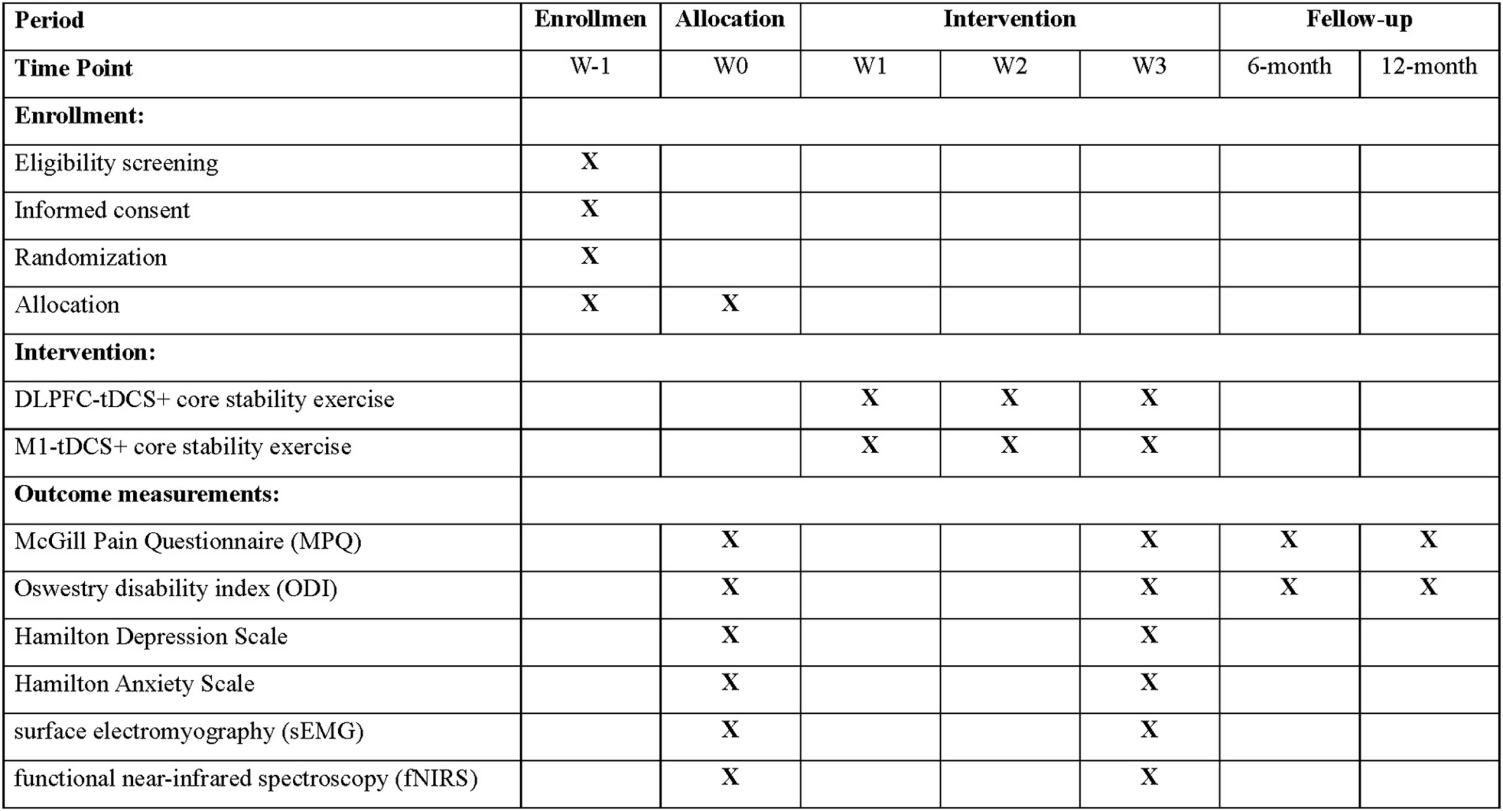
Schedule of participant enrolment, interventions, and assessments. DLPFC, dorsolateral prefrontal cortex; M1, primary motor cortex; tDCS, transcranial direct current stimulation.

The study will follow a double-blind design, where both participants and evaluators remain unaware of group assignments until the study concludes (Figure 2). Concealed allocation will be ensured using sequentially numbered, opaque, sealed envelopes. The evaluator, trained in study procedures, will be blinded to the group assignments. Only the researcher responsible for administering the tDCS intervention will have access to group allocation. A blinding scale will be applied to both the evaluators and participants to assess the effectiveness of blinding. Furthermore, to maintain blinding for participants, they will not have access to the display interface of the stimulation intervention paradigm. According to the modified Jadad scale, this blinding belongs to a high-quality blind design.

### 2.7 Masking

For practical reasons, research team members involved in delivering or supporting the interventions, including the principal investigator (PI), will not be masked. However, those responsible for conducting pre- and post-intervention assessments will remain blinded. Moreover, individuals involved in data collection and analysis will not participate in the intervention process to minimize the potential for bias.

### 2.8 Intervention

**Figure 3:**
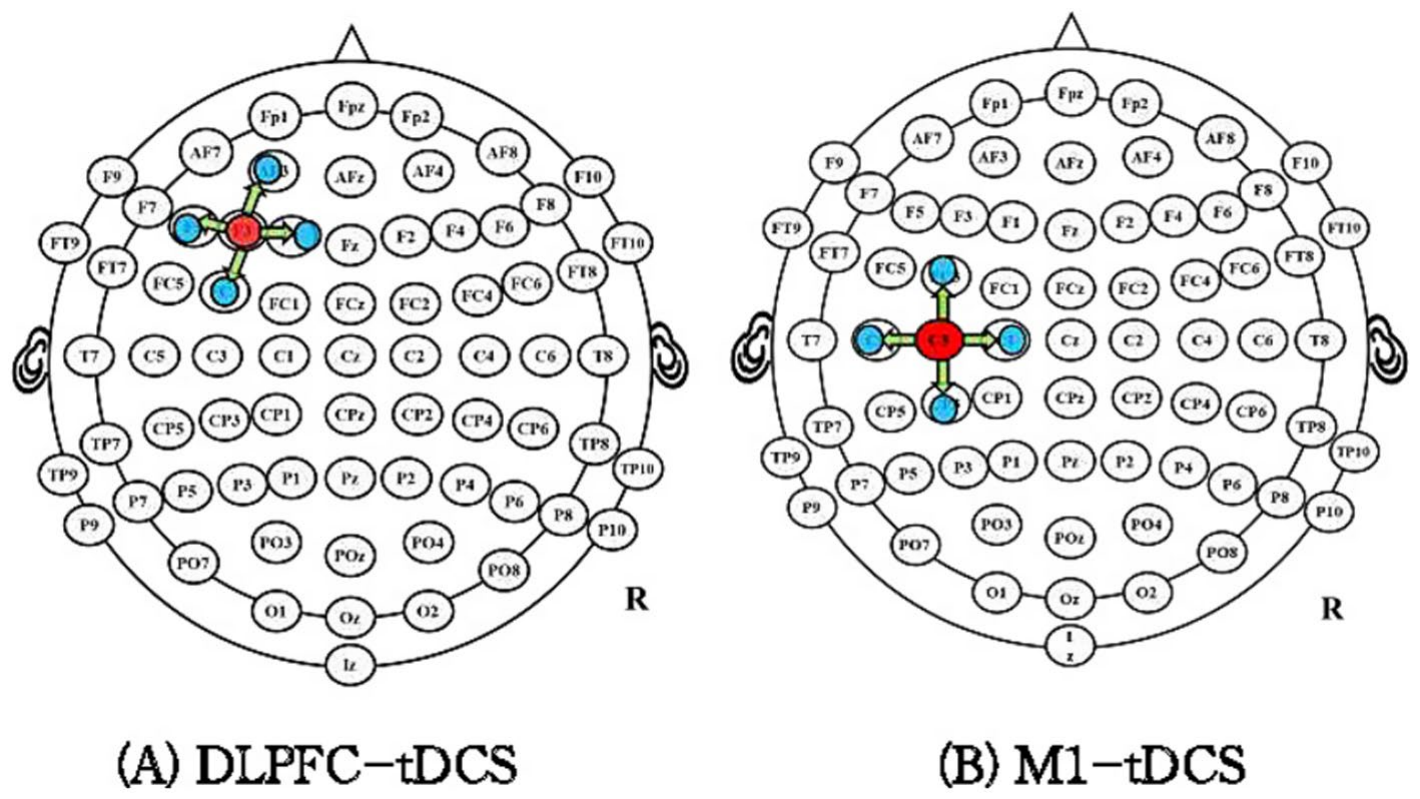
Electrode position of DLPFC-tDCS group **(A)** and M1-tDCS group **(B)**.

The international EEG 10–20 standard localization method will be used for electrode placement. In the DLPFC-tDCS group, the anode will be positioned over the left DLPFC (F3), as previous studies have shown that this region plays a key role in inhibitory control of pain processing and shows increased activity in cLBP patients (Asada et al., 2022; Cao et al., 2022). Four cathodes will be placed at a 3.5 cm radius around F3, with a current density of approximately 0.88 mA/cm^2^ (Figure 3A).

For the M1-tDCS group, the anode will be placed on the contralateral side of the most painful region, while four cathodes will be positioned at a 3.5 cm radius around C3/C4 (Figure 3B). The stimulation intensity will be gradually increased to 2 mA over the first 30 s, with a total intervention duration of 20 min per session. Each patient will undergo tDCS treatment once per day, 4 days a week, for 3 weeks.

In addition to tDCS, all participants will undergo a sling exercise program. Evaluations will be performed in four positions: prone lumbar setting, supine lumbar setting, side-lying hip abduction setting, and side-lying hip adduction setting, with weak muscle chains identified (2–3 per participant). Following the principle of progressive training, sling exercise postures—including prone bridging, supine pelvic lift, side-lying hip adduction, and side-lying hip abduction —will gradually increase in difficulty while reducing external support (as detailed in Table 1). Participants will not take analgesics or undergo any other treatments during the intervention. No pain or discomfort is expected during the sessions. The sling exercise program will be supervised by a physiotherapist with over 5 years of clinical experience. The treatment will be given once daily, 4 days per week, for 3 weeks, with 12 sessions in total.

**Table 1:** Description of sling exercise protocol.

Exercise	Exercise set up	Instruction to individual
Supine pelvic lift	Individual supine with arms parallel to body; one leg flexed with knee at 90 degree and foot on surface; narrow sling at flexed knee; Wide sling under pelvis, attached with elastic cords.	Extend knee in sling; bring another leg up parallel to other; Lift a leveled pelvis up to a straight body position.
Prone bridging	Individual prone with upper body supported on forearms; elbows directly under shoulders; narrow sling just below knee; wide sling under abdomen, attached with elastic cords.	Lift another leg from surface; lift a leveled pelvis up to a straight body position.
Side-lying hip abduction	Individual side-lying with upper body supported on shoulder; top arm parallel to body; narrow sling at knee of bottom leg; wide sling under hip, attached with elastic cords.	Lift top leg; extend bottom hip; lift up to a straight body position by pressing bottom leg into sling.
Side-lying hip adduction	Individual side-lying with upper body supported on shoulder; top arm parallel to body; narrow sling at knee of top leg; wide sling under hip, attached with elastic cords.	Lift top leg; extend bottom hip; lift up to a straight body position by pressing bottom leg into sling.

### 2.9 Data collection

A blinded researcher will gather outcome data before randomization (baseline), post-intervention (3 weeks post-randomization), and during follow-up evaluations at 6 and 12 months after the intervention. Assessments will be conducted again at the post-intervention stage to evaluate immediate effects and during follow-up to assess both short- and long-term intervention outcomes. All data will be systematically documented and stored in the trial database throughout the study. Furthermore, participants will be contacted by a designated researcher *via* telephone for follow-up. If a participant fails to complete the scheduled evaluation within 2 days of the appointment, they will receive a reminder message or a follow-up call from the research team.

### 2.10 Outcome assessment

#### 2.10.1 Primary outcome measures

##### 2.10.1.1 Pain intensity

Pain intensity will be assessed using the VAS, which ranges from 0 to 10 cm, where “0 cm” indicates no pain and “10 cm” represents unbearable pain. Moreover, the Short-Form McGill Pain Questionnaire (SFMPQ) will be used to evaluate each participant’s pain experience (Strand et al., 2008). The SFMPQ consists of 15 items divided into two categories: affective scores (4 items) and sensory scores (11 items). Each item is rated on an intensity scale from 0 to 3 (0 = none, 1 = mild, 2 = moderate, 3 = severe). Pain assessments will be conducted at all time points, including baseline, 3 weeks, 6 months, and 12 months post-randomization. Meanwhile, during the follow-up period, we will record whether the patients are using painkillers or other treatments.

##### 2.10.1.2 Disability

Disability associated with cLBP will be measured using the Oswestry Disability Index (ODI), a validated tool consisting of 10 questions, each with 6 response options scored from 0 to 5. This index evaluates functional impairment in cLBP by incorporating pain and physical activity measures. Based on the total score, disability is categorized as bed-ridden (81–100%), crippled (61–80%), severe disability (41–60%), moderate disability (21–40%), or minimal disability (0–20%) (Martí-Salvador et al., 2018). Participants will be instructed to complete the questionnaire based on their condition on the day of assessment.

##### 2.10.1.3 Mental health

Mental health will be assessed using the short version of the Hamilton Depression Scale (HDS) and the Hamilton Anxiety Scale (HAMA). The HDS consists of three subscales, each comprising seven items, designed to evaluate anxiety, depression, and stress over the previous week (Kaptan et al., 2012). Responses are rated on a scale from 0 to 3 (fully disagree to fully agree), with total scores indicating symptom severity: a score of 13 or higher suggests depressive symptoms, 14–27 indicates mild depression, 28–41 reflects moderate depression, and scores of 42–53 suggest high level depression. The HAMA is primarily used to evaluate the severity of anxiety symptoms in individuals with neurosis and other conditions. It evaluates 14 aspects of anxiety, including mood, depressive mood, cognitive function, insomnia, fear, tension, somatic anxiety, reproductive and urinary symptoms, gastrointestinal and digestive symptoms, respiratory symptoms, cardiovascular symptoms, sensory symptoms, and behavioral responses during social interactions (Yin et al., 2023).

#### 2.10.2 Secondary outcome measures

##### 2.10.2.1 Muscle synergy

Muscle synergies will be assessed using wireless surface electromyography (sEMG) (Trigno, Delsys, Inc., USA). To optimize electrode conductivity, thorough skin preparation will be performed before electrode placement. The muscle activity of the erector spinae (ES), multifidus (MF), rectus abdominis (RA), and bilateral transversus abdominis (TA) muscles will be recorded (as shown in Table 2). Electrode placement will follow the standardized guidelines recommended in previous studies (Hermens et al., 2000; Park et al., 2014). The extraction of muscle synergies will be carried out using non-negative matrix factorization (NNMF) and principal component analysis (PCA) (Bekius et al., 2020; Rabbi et al., 2020; Wang et al., 2015). The analysis will include measurements of the muscle synergies number, complexity, sparseness, clusters, and muscle networks.

**Table 2:** Placement of the sEMG electrodes.

Muscle	Electrode placement location
Transversus abdominis (TA)	Along either side of the course of the underlying muscle fibers and centered 2 cm cephalic to the pubic bone, just lateral to the midline, and parallel to the superior pubic ramus
Rectus abdominis (RA)	2 cm lateral from the midline of the umbilicus
Multifidus (MF)	A line from caudal tip posterior spinal iliac superior to the interspace between L1 and L2 interspace at the level of L5 spinous process (i.e., about 2–3 cm from the midline)
Erector spinae (ES)	2 cm lateral to the L3 level

##### 2.10.2.2 Concentration of oxygenated and deoxygenated hemoglobin in the cerebral cortex

**Figure 4:**
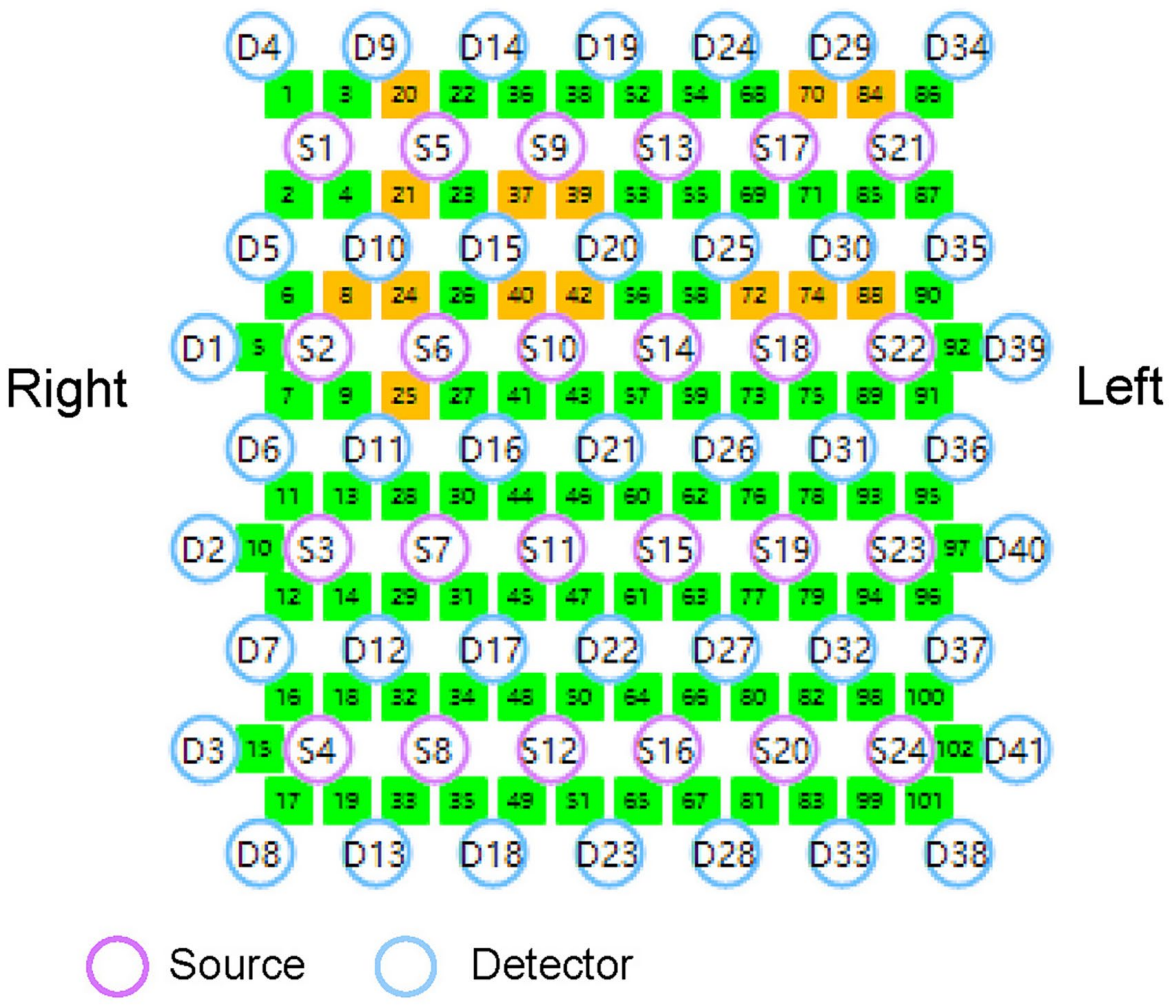
The fNIRS channels arrangement across the cerebral cortex.

A multichannel fNIRS system (Nirsmart, Danyang Huichuang Medical Equipment Co., Ltd., China) will be used for continuous measurement and recording of changes in oxygenated hemoglobin (HbO) and deoxyhemoglobin (HbR) concentrations during task performance. The system consists of a near-infrared light source and avalanche photodiode detectors, operating at wavelengths of 730 nm and 850 nm, respectively. It has a sampling rate of 24 light sources and 41 detectors, forming 102 effective channels (Figure 4). The average source-detector distance is 3 cm (range 2.7–3.3 cm). Following the international 10–20 system for electrode placement, this study will primarily examine brain activity in the cerebral cortex of the M1 and DLPFC. The probe locations, positioned at Ar, Al, Cz, Nz, and Iz according to the 10–20 system, will be measured using an electromagnetic 3D digitizer (Patriot, Polhemus, VT, USA) on a model head. The obtained grand-averaged coordinates will then be processed with NirSpace (Danyang Huichuang Medical Equipment Co., Ltd., China) to calculate Montreal Neurological Institute (MNI) coordinates, identify the associated brain regions, and calculate the channels overlap probability (Tsuzuki et al., 2007). Further details regarding brain regions and channel distribution are provided in Supplementary material.

##### 2.10.2.3 Task performance

When conducting sEMG and fNIRS assessment, the subjects performed the following tasks. Task 1: The task of quickly raising arms with both hands. Task 2: Dual-task cognitive test. Complete the dual tasks of quickly raising the left/right arm or taking steps with the left/right leg based on the Flanker task paradigm. Task 3: Balance ability assessment, left/right single-leg standing and unbalanced standing.

### 2.11 Data analysis

#### 2.11.1 Statistical analysis

Statistical analyses will be performed using SPSS (version 25.0, IBM, USA). Before performing descriptive statistics, the Shapiro–Wilk test will be applied to evaluate the normality of data distribution. Data following a normal distribution will be reported as mean and standard deviation (SD), while non-normally distributed data will be expressed as medians with interquartile range (IQR). Categorical variables will be summarized as percentages and frequencies. Baseline demographic and clinical characteristics will be compared between groups using appropriate statistical tests based on the data type. Continuous variables that follow normal distribution will be analyzed while using independent sample *t*-tests, whereas non-parametric Mann–Whitney *U*-tests will be applied for non-normally distributed continuous data. Categorical variables will be analyzed using chi-square tests or Fisher’s exact tests, as appropriate. Correlations between variables will be evaluated using the Pearson correlation coefficient or Spearman correlation coefficient based on the type of data. A repeated-measures analysis of variance (2 groups × 4 time points) will be performed to assess the therapeutic effects on primary and secondary outcomes. If a significant main effect was observed, *post hoc* tests with Bonferroni correction were conducted. A *p*-value of <0.05 will be considered statistically significant.

#### 2.11.2 sEMG data analysis

To process the sEMG signals, a high-pass filter with a cut-off frequency of 20 Hz will be applied, followed by rectification using the Hilbert transform to generate sEMG envelopes (Allen et al., 2020). The envelopes obtained from repeated trials will be averaged across different experimental paradigms and used for muscle synergy and muscle network analyses. Muscle synergies will be extracted using NNMF and PCA. Both methods will be employed to determine the number of muscle synergies (Bekius et al., 2020; Boonstra et al., 2015), synergy complexity (d'Avella and Bizzi, 2005), and muscle synergy sparseness (Cheung et al., 2020). The analysis will be conducted using Matlab 2019a (MathWorks Inc., Natick, Massachusetts, USA). NNMF is a technique that decomposes a data matrix (*A*) into two non-negative matrices: a command matrix (*K*) and a synergy matrix (*W*), ensuring that all components remain non-negative. PCA, on the other hand, identifies orthogonal principal components (PCs) that represent the most significant variance within the EMG dataset (Yin et al., 2015; Jolliffe and Cadima, 2016). These PCs reflect collective muscle activation patterns and their overall contribution to data variability. Using PCA and NNMF allows for a comprehensive understanding of potential synergies across different experimental conditions. To evaluate muscle synergy complexity, the total variance accounted for by a single synergy solution (tVAF1) will be computed, as it is associated with muscle function. For assessing muscle synergy sparseness, a sparse synergy vector containing a single non-zero component will be assigned a sparseness value of 1, whereas a non-sparse synergy vector, in which all muscles contribute equally, will be assigned a sparseness value of 0 (Cheung et al., 2020). Furthermore, significantly active muscles will be identified based on a loading coefficient exceeding 0.5 within a given PC. The frequency of significantly activated muscles for each PC will be analyzed based on different experimental paradigms. Muscle network analysis will also be performed using intermuscular coherence and partial directed coherence (PDC) to evaluate functional connectivity among muscles. The HERMES toolbox will be used to estimate connectivity between different sEMG envelopes (Boonstra et al., 2015). Coherence and PDC analyses will be conducted to assess undirected and directed connectivity patterns.

#### 2.11.3 fNIRS data analysis

The NirSpark software package will be employed to preprocess fNIRS data. Initially, raw fNIRS signals will be transformed into optical density changes by applying a logarithmic transformation. Channels with excessively low optical intensity (coefficient of variation > 10%) will be removed to eliminate noise. A bandpass filter will be applied to minimize physiological artifacts and instrumental noise, with high-pass and low-pass cut-off frequencies set at 0.01 Hz and 0.1 Hz, respectively. Changes in [HbR] and [HbO] concentrations will then be determined using the modified Beer–Lambert law. To analyze cerebral cortical activity during tasks, a general linear model (GLM) will be used, represented by the equation:


Δ[Hbx]=X×β+ε

where Y represents the variation in [HbR] or [HbO] concentration, X denotes the modeled neural response predicted based on task onset and convolved with a classical hemodynamic response function, β is the regression coefficient reflecting the activation level of each channel, and ɛ accounts for unexplained variability or noise in the data. The β-value (μmol), which quantifies activation at each channel, will be calculated alongside the standard errors for [HbR] and [HbO] in both groups for each trial.

### 2.12 Data collection, management, and monitoring

Data collection will be performed by experienced attending physicians, therapists, and researchers who are independent of the treatment allocation and intervention procedures. All collected data will be promptly recorded in designated case report forms (CRFs). A designated researcher will digitize the data while ensuring patient privacy and data security through unique identifiers. Two data managers, who will remain blinded to group assignments, will independently process the completed CRFs and enter the data into an Excel database. To maintain data integrity, both data managers will undergo comprehensive training in data monitoring and entry procedures.

To reduce the probability of an adverse event, the participants will be rigorously screened in accordance with the exclusion and inclusion criteria. Adverse effects and events will be closely monitored during the clinical trial. If this occurs, it will be noted in the CRF. Some incidents will be monitored, and when these incidents are inappropriate, the treatment will be discontinued, such as exacerbation of the condition, serious adverse events, poor adherence leading to a loss of follow-up or the development of a new serious illness affecting the course of this protocol.

### 2.13 Quality control

The steering committee will oversee quality control throughout the trial. Before their involvement in the study, all researchers will receive training in professional trial methods and routine monitoring techniques to ensure methodological consistency. If any modifications or corrections are made to the study protocol, the steering committee and ethics committee will be notified accordingly. Regular monitoring will be conducted to ensure adherence to the study protocol and the integrity of collected data.

## 3 Discussion

### 3.1 Summary

This study presents the protocol for an RCT designed to assess the efficacy of tDCS targeting the DLPFC in patients with cLBP. The trial aims to determine whether DLPFC-tDCS can improve activity and functional connectivity between the M1 and DLPFC. The results of this study will provide critical insights into the potential therapeutic role of DLPFC-tDCS in improving M1-DLPFC connectivity and trunk muscle synergy, forming a theoretical foundation for novel treatment approaches for cLBP. Previous research has used multivariate pattern analysis and fMRI to identify disruptions in functional connectivity among key brain networks in cLBP patients. These networks include salience, sensorimotor, the default mode, and central executive networks. Findings suggest that the rostral anterior cingulate cortex and medial prefrontal cortex may serve as critical hubs associating the default mode network with other neural circuits in individuals with cLBP (Tu et al., 2019). Furthermore, a study published in *NeuroImage* demonstrated that the DLPFC has extensive anatomical projections to M1 and that these two regions interact closely during motor tasks. This suggests that exercise-induced neuroplasticity may result from altered input from the DLPFC to M1 rather than the training effect within M1 itself (Cao et al., 2022). These findings highlight the relevance of M1-DLPFC functional connectivity in motor control and muscle coordination.

To the best of our knowledge, this study will be the first to investigate the pathological mechanisms of cLBP by examining brain connectivity and trunk muscle synergy. We hypothesize that the abnormal coordination patterns observed in trunk muscles in cLBP patients are not only a result of adaptive changes within M1 and DLPFC but also due to reduced functional connectivity between these regions. This connectivity impairment may play a key role in the neuromuscular dysregulation of trunk muscles in cLBP. By comparing the therapeutic effects of M1-tDCS and DLPFC-tDCS, we expect that M1-tDCS will primarily increase M1-supplementary motor area (SMA) connectivity, while DLPFC-tDCS will strengthen M1-DLPFC functional connectivity. This improvement in connectivity is expected to restore trunk muscle coordination and improve clinical symptoms.

### 3.2 Strengths and limitations

One of the major strengths of this protocol is that it represents one of the first clinical trials investigating the efficacy of DLPFC-targeted tDCS in patients with cLBP. If proven effective, DLPFC-tDCS could serve as a viable therapeutic option for managing cLBP. Another advantage of the study is its potential to reveal the neuroregulatory mechanisms underlying the functional connections between brain regions and their impact on trunk muscle coordination in cLBP patients.

However, there will be some limitations. A key limitation is the absence of a healthy control group. Including healthy participants would allow for a more comprehensive comparison with the M1-tDCS and DLPFC-tDCS groups, improving the interpretation of the results. Moreover, differences in the sampling rates of the two fNIRS systems used during measurements present a methodological challenge. Since the sampling rates of fNIRS and sEMG differ, direct correlation analyses between their signals cannot be performed. Instead, only data-level analyses can be conducted, which may limit the ability to fully capture the relationship between cortical activity and muscle activation patterns.
